# *In vitro* and *in vivo* degradation, biocompatibility and bone repair performance of strontium-doped montmorillonite coating on Mg–Ca alloy

**DOI:** 10.1093/rb/rbae027

**Published:** 2024-03-22

**Authors:** Wenxin Sun, Kaining Yang, Yuhong Zou, Yande Ren, Lin Zhang, Fen Zhang, Rongchang Zeng

**Affiliations:** Department of Bioengineering, College of Chemical and Biological Engineering, Shandong University of Science and Technology, Qingdao 266590, China; Department of Bioengineering, College of Chemical and Biological Engineering, Shandong University of Science and Technology, Qingdao 266590, China; Department of Bioengineering, College of Chemical and Biological Engineering, Shandong University of Science and Technology, Qingdao 266590, China; Affiliated Hospital of Medical College Qingdao University, Qingdao 266555, China; Hospital of Shandong, University of Science and Technology, Qingdao 266590, China; Corrosion Laboratory for Light Metals, College of Materials Science and Engineering, Shandong University of Science and Technology, Qingdao 266590, China; Corrosion Laboratory for Light Metals, College of Materials Science and Engineering, Shandong University of Science and Technology, Qingdao 266590, China

**Keywords:** Mg–Ca alloy, montmorillonite, strontium, corrosion resistance, biocompatibility, osteogenesis

## Abstract

Poor bone growth remains a challenge for degradable bone implants. Montmorillonite and strontium were selected as the carrier and bone growth promoting elements to prepare strontium-doped montmorillonite coating on Mg–Ca alloy. The surface morphology and composition were characterized by SEM, EDS, XPS, FT-IR and XRD. The hydrogen evolution experiment and electrochemical test results showed that the Mg–Ca alloy coated with Sr-MMT coating possessed optimal corrosion resistance performance. Furthermore, *in vitro* studies on cell activity, ALP activity, and cell morphology confirmed that Sr-MMT coating had satisfactory biocompatibility, which can significantly avail the proliferation, differentiation, and adhesion of osteoblasts. Moreover, the results of the 90-day implantation experiment in rats indicated that, the preparation of Sr-MMT coating effectively advanced the biocompatibility and bone repair performance of Mg–Ca alloy. In addition, The Osteogenic ability of Sr-MMT coating may be due to the combined effect of the precipitation of Si^4+^ and Sr^2+^ in Sr-MMT coating and the dissolution of Mg^2+^ and Ca^2+^ during the degradation of Mg–Ca alloy. By using coating technology, this study provides a late-model strategy for biodegradable Mg alloys with good corrosion resistance, biocompatibility. This new material will bring more possibilities in bone repair.

## Graphical abstract

**Figure:**
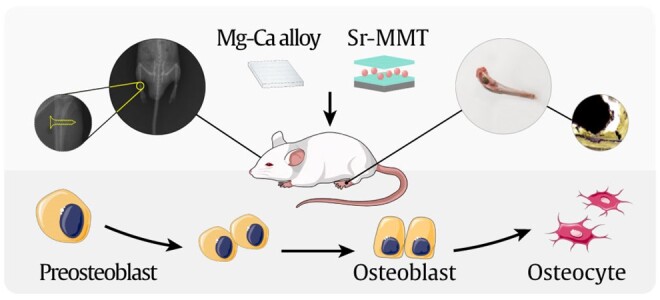

## Introduction

The need for bone repair materials is rising as the global population ages and the prevalence of bone and joint disorders rises. Mg could facilitate bone regeneration by proliferating osteoblasts, promoting osteogenic differentiation and achieving mineralization [1]. Compared with autologous bone and allogeneic bone, magnesium and its alloys have attracted attention due to their good mechanical properties and biocompatibility [2–4]. However, the AZ31 magnesium alloy that has been industrially produced is controversial because it contains Al^3+^ ions that are harmful to the human body[5, 6]. Therefore, it is necessary to choose magnesium alloys that release ions that are harmless to the human body. Calcium is a vital element in the composition of human bones, of which 99% of the calcium in the human body exists in bones and teeth. Because it has a density similar to that of human bones (1.55 g/cm^3^), it is an ideal material for the elements contained in magnesium alloys [7–11]. However, magnesium alloy, as a biodegradable metal, degrades too fast in the human environment, which makes it insufficient to support patient recovery and seriously affects its clinical application [12–14].

One efficient strategy to increase the magnesium alloy’s resistance to corrosion and biocompatibility is surface modification [15–17]. At present, micro-arc oxidation, electrochemical deposition, and magnetron sputtering are commonly used as methods for preparing coatings on surfaces [18–24]. However, these methods are technically complex and expensive, so people are also making more convenient and economical coating preparation methods. The hydrothermal method is a promising coating preparation method due to its simple structure, economical and easy preparation of composite coatings [25, 26]. A suitable coating preparation method is only the first step. For implant materials, the ideal coating material should be nontoxic and well biocompatible [27, 28]. At present, there are many kinds of coatings used on the surface of magnesium substrate. To increase the magnesium alloy’s resistance to corrosion, Sun *et al*. [29] prepared a composite coating of poly L-lactic acid and magnesium aluminum-layered double hydroxide(LDH/PLLA-10) on the surface of AZ31. Compared with magnesium alloy substrate, the icorr density of LDH/PLA-10 coating decreases by three orders of magnitude and has the lowest hydrogen evolution rate (HER). Zhang *et al.* [30] prepared calcium phosphate coating induced by layer by layer assembly of polyvinylpyrrolidone and DNA(Ca-P(PVP/DNA)_20_) on the surface of AZ31, and the corrosion current density of Ca-P(PVP/DNA)_20_ changed from 1.63 × 10^−5^ A·cm^−2^ to 29 × 10^−6^ A·cm^−2^. The calcium phosphorus coating induced by glucose and L-cysteine biomimetic Schiff base prepared by Wang *et al.* [31] on the surface of AZ31 also improves the impedance of magnesium alloy. Among them, because of Ca and P elements make up the majority of bone, bioactive calcium phosphate (Ca-P) coatings have attracted much attention [32, 33]. Su *et al.* [34] studied the preparation process of calcium phosphate conversion coating (CPCC) and determined that the pH value and temperature of the reaction significantly affect the coating structure and porosity, which can affect the corrosion performance of CPCC coatings. It is worth noting that Guo *et al.* [35] looked at more than just how calcium phosphorus coating affected magnesium alloys’ ability to withstand corrosion, and studied the biocompatibility of Ca-P coating through *in vitro* experiments. The experimental results indicate that Ca-P coating can significantly increase the magnesium alloys’ resistance to corrosion and biocompatibility, providing a theoretical basis for the application of Ca-P as a coating material in medical magnesium alloys. In order to make the Ca-P coating have more additional properties, the Ca-P coating is often combined with other substances. Cui *et al*. [36] and Zhang *et al*. [37] prepared Ca-P coating induced by polyacrylic acid multilayer and Ca-P coating induced by sodium copper chlorophyllin with porphyrin ring on the surface of AZ31 through layer by layer assembly technology. These two coatings improved the corrosion resistance and biocompatibility of magnesium alloy, and made it possessed antibacterial properties. The research of Ca-P coating as coating material emerges one after another, however, there are few articles about montmorillonite (MMT) as coating material [38]. The layered mineral MMT is made up of hydrous aluminosilicates with incredibly tiny grains, it with an aluminum-oxygen octahedron in the middle and a three-layer sheet-like structure composed of silicon-oxygen tetrahedrons up and down. Its higher ion exchange capacity and higher water absorption and expansion capacity comes from the water and certain exchange cations between the layers of the crystal structure, and it has good biocompatibility and has been widely used in applications such as adsorbents and drug delivery vehicles [39–43]. However, MMT coating alone cannot meet the needs of patients with bone defects, and other substances need to be added on this basis to promote bone repair. Our research team has previously studied the performance of gentamycin MMT coating and albumin MMT coating on the surface of AZ31 substrate [44, 45]. The results displayed that the addition of gentamicin and albumin enhanced the antibacterial property and biocompatibility of magnesium alloy. However, these two studies did not mention the research of coating on improving the repair performance of matrix bone. Therefore, the current research direction of our research group is to find a material with bone repair ability to combine with MMT to prepare a composite coating.

For the human body, strontium is one of the indispensable trace elements. It exists in various tissues of the human body. It is an important component of teeth and bones and is closely related to the formation of human bones [46]. According to studies, strontium is an ideal element for boosting bone repair since it inhibits osteoclasts and promotes osteogenesis [47, 48]. Among them, strontium ranelate has become the main drug for clinical treatment of osteoporosis. At present, in the aspect of bone materials, the application research of strontium mainly involves alloying, bioactive glass, strontium-containing coating and bone tissue engineering materials [49–52].

In this research, strontium-doped MMT (Sr-MMT) was prepared by using the ion exchange properties of MMT, and a bone implant material with bone repair performance was prepared with magnesium–calcium (Mg–Ca) alloy as the matrix. The research aims to address the issues of rapid degradation and poor bone growth of Mg–Ca alloys after implantation *in vivo* by improving their corrosion resistance and enhancing their ability to promote bone growth. Investigations were conducted on the Sr-MMT coatings’ surface shape, chemical composition, corrosion resistance, and *in vitro* compatibility. Furthermore, *in vivo* degradation and biocompatibility studies of Sr-MMT coatings were performed in rat models implanted for 1 and 3 months, respectively.

## Materials and methods

### Ethical approval

All animal experiments were conducted according to the ISO 10993-2:1992 animal welfare requirements. The protocol for their care and the use of laboratory animals was approved by the Animal Ethical Committee of the Affiliated Hospital of Qingdao University (approval date: October 24, 2023; approval number: QYFY WZLL 32751).

### Materials and chemicals

Extruded Mg–Ca alloy was utilized in this work. After polishing the samples using sandpaper up to 1200 grit, they were cleaned of surface impurities using acetone and ethanol before being dried for further usage. Na-MMT powder, strontium nitrate (Sr (NO_3_)_2_, analytical reagent, 99.0%) and all substances for Dulbecco’s-modified eagle medium (DMEM) were purchased by QingDao Jingke Chemical Reagent Co. Ltd, China.

#### Preparation of Na-MMT and Sr-MMT coatings

Sr-MMT powder was prepared by ion exchange of Na-MMT powder and strontium nitrate in deionized (DI) water. Five grams of Na-MMT powder and 95 ml DI water were placed in a 250-ml beaker and stirred at room temperature for 5 h. Then 0.85 g Sr (NO_3_)_2_ was added and stirred with them for 4 h at 70°C and pH7. The suspension was centrifuged at 4000 rpm for 15 min and washed repeatedly with DI water. The resulting precipitate was dried at 70°C for 36 h and then ground and sieved.

**Figure 1.**
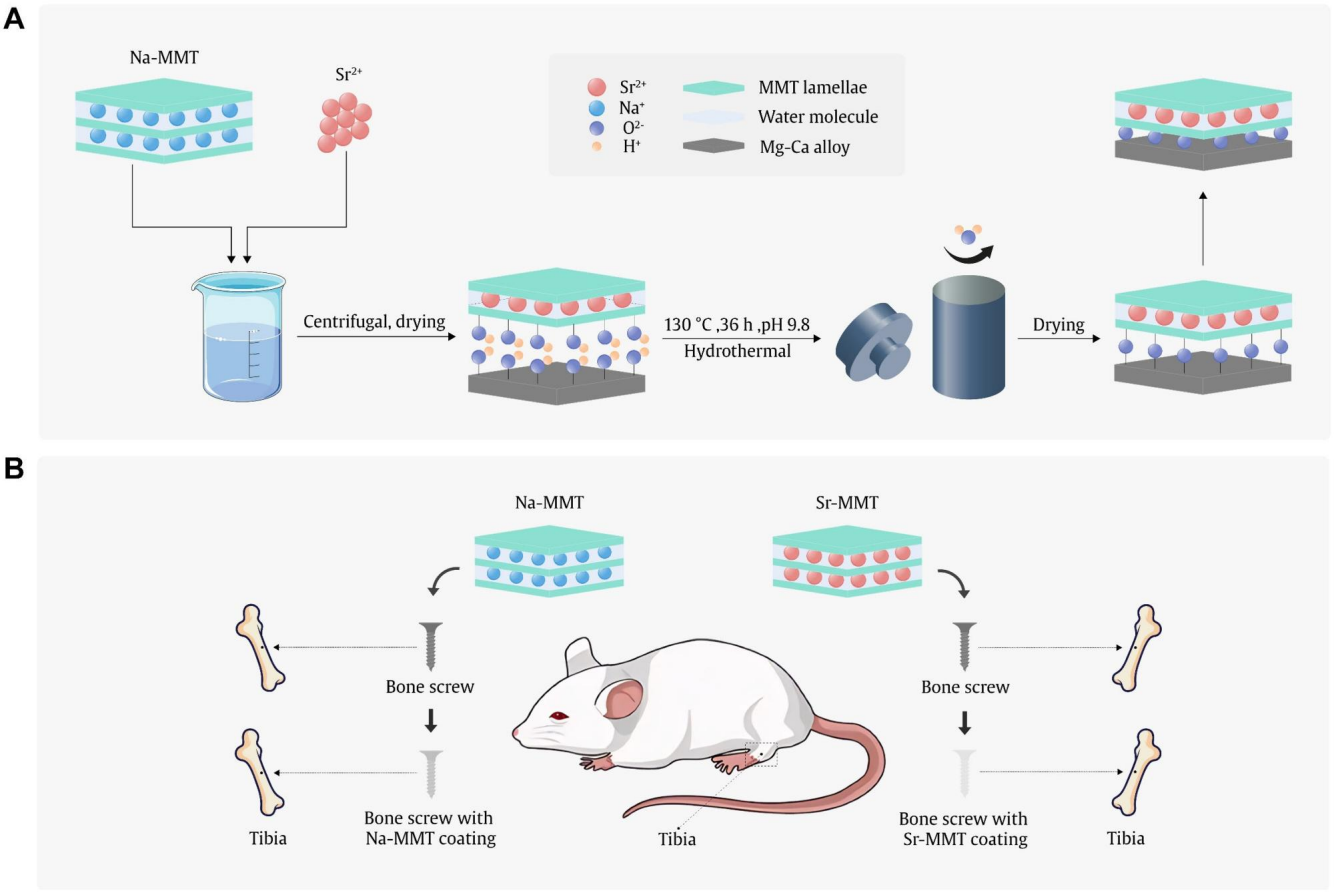
(**A**) Preparation process of Sr-MMT coating. (**B**) Establishment of bone implantation model.

The Sr-MMT powder was mixed with DI water at 3% by mass and stirred at room temperature for 5 h. Then it was stirred for 5 h at 80°C, during which the pH was adjusted twice to control the suspension at a pH of 9.8. The suspension and the polished Mg–Ca alloy samples were placed in an autoclave and heated in an oven at 130°C for 36 h. The Sr-MMT coating was prepared on the surface of the Mg–Ca alloy by a hydrothermal method. The above process is shown in Figure 1A, the preparation method of the Na-MMT coating is the same as the above steps.

#### Surface characterization

The surface morphology and thickness of Sr-MMT and Na-MMT coatings were observed by means of scanning electron microscope (SEM, Nova NanoSem 450) equipped with energy dispersive X-ray spectroscopy (EDS). The chemical bonds of the coatings were observed through Fourier transform infrared spectroscopy (FT-IR, Nicolet 380, Thermoelectron, USA). X-ray photoelectron spectroscopy (XPS, Thermo Scientific K-Alpha, USA) with mono AlK_*α*_ radiation (hv = 1486.6 eV) was performed to analyze chemical composition of Sr-MMT and Na-MMT coatings.

The Micro Combi Tester (Anton Paar MCT and NHT, Austria) was used to evaluate binding force between Sr-MMT coating and Mg–Ca alloy matrix. The loading rate is 1.99 N·min^−1^, and the load range is 0.01 N ∼ 2 N. Scratch images are obtained by *in situ* optical microscope to determine where the coating failed.

### Corrosion behavior

To research whether the preparation of coatings improves the corrosion resistance of Mg–Ca alloys, the corrosion resistances of Sr-MMT and Na-MMT properties were tested.

#### Electrochemical corrosion tests

Electrochemical impedance spectroscopy (EIS) and potentio-dynamic polarization curves were conducted on electrochemical workstation (VersaSTAT 4, Princeton, USA) at a scan rate of 1.0 mV/s in DMEM at 37°C. The samples with an area of 1 cm^2^ exposed to DMEM solution were used as the working electrodes, the platinum electrode was used as the auxiliary electrode, and the saturated calomel electrode (SCE) was used as the reference electrode.

#### Immersion corrosion tests

The corrosion resistance of the bare alloy, Na-MMT- and Sr-MMT-coated Mg–Ca alloys were tested in DMEM with pH 7.4 at 37°C for 2 weeks. Meanwhile, the ratio of sample surface area to DMEM solution volume was 6.9:250 cm^2^·ml^−1^. During the first 12 h of immersion, the pH changes of the DMEM solution and the volume changes of hydrogen in the burette were monitored every 1 h. The formula for calculating the HER (ml·cm^−2^·h^−1^) is:

(1) $$V_{H}=V/s\cdot t$$

where *V*_H_ means HER; *V* is hydrogen evolution volume (ml); s is the area of the sample exposed to the solution (cm^2^), and *t* means immersion time of sample in solution (h).

Three sets of parallel experiments were set up.

### *In vitro* study

#### Cytotoxicity assay

##### CCK-8 assay

CCK-8 method was used to test the changes of cell viability when MC3T3-E1 cells were co-cultured with samples for 24 and 72 h. MC3T3-E1 cells were cultured in a 5% CO_2,_ 37°C incubators. And the ratio of cell complete medium is αMEM medium: fetal bovine serum = 9:1. The samples were aseptic by ultraviolet irradiation for 30 min, and then placed in 30 ml of cell culture solution for 15 min. The collected sample leaching solutions were stored at 4 for future use. MC3T3-E1 cells in logarithmic growth phase were taken and seeded into 96-well plates at 6 × 10^3^ cells/well and co-cultured with the sample extract. Culture conditions were 37°C and 5% CO_2_. Following the 24- and 72-h cultures, the medium was withdrawn, and each well of the plate was washed 3 times with phosphate-balanced solution (PBS). Then added 100 μl/well of medium containing 10% CCK-8, and incubated for 2 h. Microplate reader (TECAN, SPARK 10M) detected absorbance at 450 nm. The formula for calculating relative cell viability is as follows:

(2) $$\mathrm{Relative}\;\mathrm{viability}\;(\%)\;=\frac{OD_{E}-OD_{B}}{OD_{C}-OD_{B}}\times 100\%$$

where OD_E_ is the absorbance value of experimental well, OD_C_ is the absorbance value of control well and OD_B_ is the absorbance value of blank well.

##### Live/dead cell staining

MC3T3-E1 cells were cultured with samples at a density of 1 × 10^4^ cells/well in 12-well plates. Following a 24- or 72-h culture, PBS was used to wash the combined solution. The washed cells were placed in a staining working solution prepared by mixing calcein-am and propidium iodide (PI) in a certain proportion, incubated at 4°C in the dark for 15 min, and then washed with PBS. The results were observed with a laser confocal microscope (Leica TCS SP8, Leica).

### Osteogenic performance assay

The MC3T3-E1 cells in the logarithmic growth phase were taken and inoculated into special confocal culture dish according to 6 × 10^4^ cells/dish, and cultured in a 5% CO_2_, 37°C constant temperature incubator for 24 and 72 h. In addition, inoculated cells into a 12-well plate with 30 × 10^4^ cells per well and incubated for 72 h in a constant temperature incubator with 5% CO_2_ and 37°C. The culture medium was removed, the cells were washed with PBS and scraped off, and 200 μl of PBS was added to collect the cell suspension. After the homogenate was broken, centrifuged it at 4°C for 10 min, and the supernatant was taken for detection. OD value measured by microplate reader (TECAN, SPARK 10M) at 405 nm. After aspirating the culture medium, wash the cells twice with 1×PBS (pH = 7.4) pre-warmed at 37°C. Cells were fixed for 10 min at room temperature in a 4% formaldehyde solution in PBS. PBS was used to wash the cells 2–3 times, and then 200 µl of 100 nM TRITC-labeled phalloidin working solution was obtained, and it was incubated for 30 min in the dark at room temperature. Cells were washed 3 times with PBS again and nuclei were counterstained with 200 µl 4,6-diamino-2-phenyl indole (DAPI) solution (concentration: 100 nM). Photographed with a laser confocal microscope (Olympus, IX73) at 200×.

### *In vivo* study

#### Establishment of bone implantation model

Establishment of bone implantation model is as shown in the Figure 1B (Bone screw material, φ2 × 8 mm, provided by Suzhou Zhuocha Medical Technology Co., Ltd). Twenty-week-old male Wistar rats (clean grade, Shandong Animal Center) were selected as experimental objects. Na-MMT and Sr-MMT coatings were prepared on the bone screw, and the bone screw implantation experiment was divided into three groups: Na-MMT group, Sr-MMT group, with five Westar rats in each group. The rats were anesthetized and prepared for skin preparation, and a 1.5-mm diameter defect was prepared at the knee joint end of each rat’s tibia with an electric drill. The defects of each rat were filled with bare alloy bone screws and coated bone screws, respectively. On the day of implantation, 1 month and 3 months later, the degradation of bone screw in rat tibia and the growth of bone tissue around bone screw were observed by X-ray.

#### Hard tissue section and staining

After 3 months of feeding, the rats were killed and their tibias were taken out. The removed tibias were not decalcified, and were directly embedded and polymerized in the light curing machine (Composition of cured resin: Methyl methacrylate, Dibutyl phthalate and Benzoyl peroxide, MACKLIN, China). Taking out the tissue block, the tissue was cut into 25 μm sections with a hard tissue slicer (Section-grind system, LANMING Xi an, China), and then hematoxylin and eosin (HE) staining were conducted. the stained sections were dehydrated and sealed for further image acquisition and analysis.

### Statistical analysis

All the data are expressed as mean±SD. Statistical analysis was performed using one-way ANOVA followed by Tukey’s test. It is considered as a statistically significant difference when *P* ≤ 0.05. Statistical analysis was conducted with SPSS 19.0.

## Results

### Surface characterization

**Figure 2.**
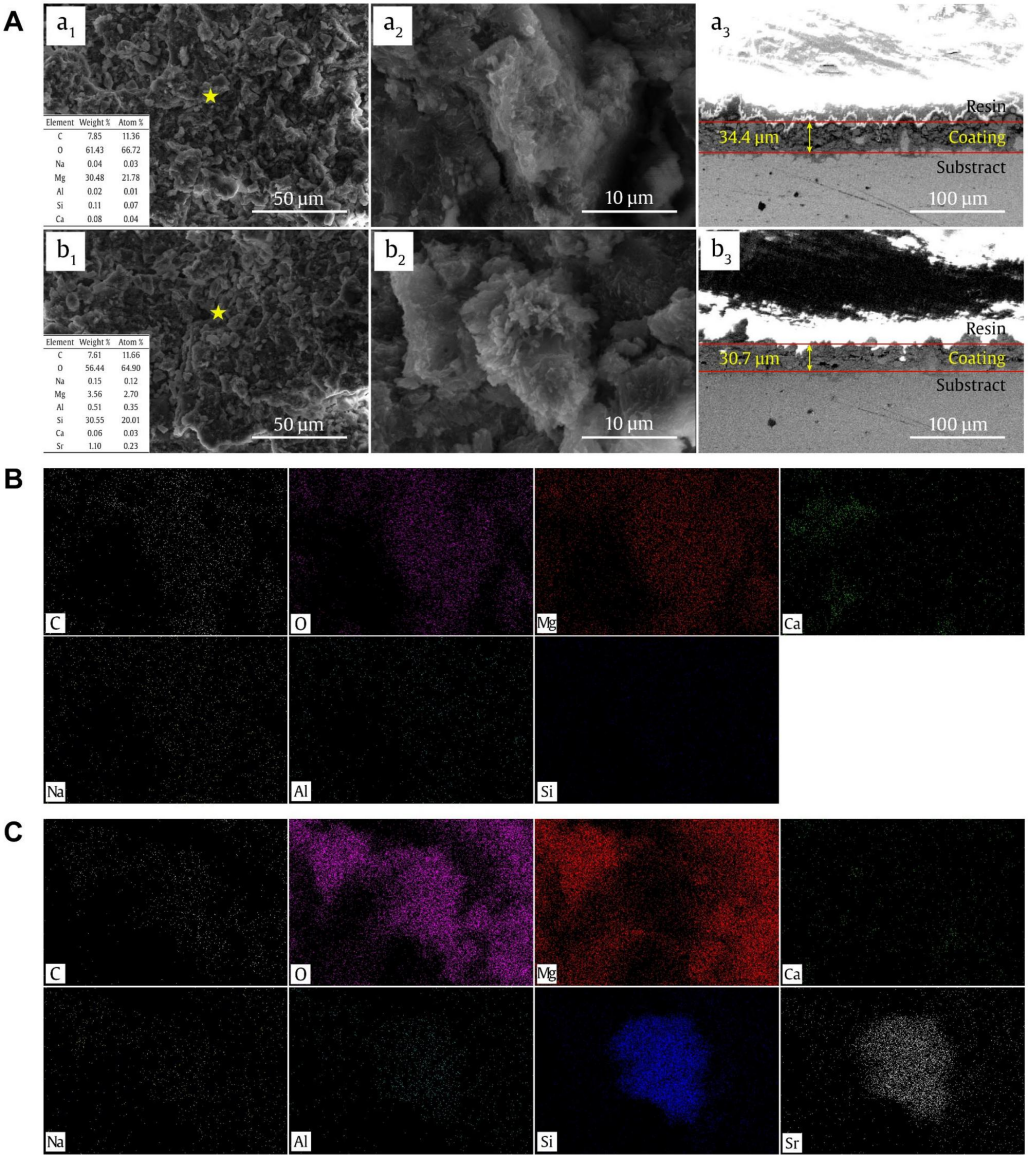
(**A**) SEM morphologies and the corresponding EDS spectra of (a_1_–a_3_) Na-MMT, and (b_1_–b_3_) Sr-MMT coating. (**B**) Mapping of the Na-MMT coating. (**C**) Mapping of the Sr-MMT coating.

The surface morphologies of Sr-MMT and Na-MMT coatings are shown in Figure 2A. It can be observed from the electron microscope that the Na-MMT and Sr-MMT coatings are successfully covered on the surface of the Mg–Ca alloy and show lamellar structure, while the Sr-MMT coating shows denser and more uniform structure than Na-MMT coating. The coatings seem to bond well with the Mg–Ca alloy. Apart from Si, Mg, Al, O, and Ca, the EDS results (Figure 2Aa1 and b1) show that Na and Sr are found on the surface of Na-MMT and Sr-MMT coatings, which means that two kinds of coatings are successfully prepared on the surface of Mg–Ca alloy. Moreover, the coating thickness of Na-MMT and Sr-MMT can be obtained by Figure 2A(a3) and (b3), it to be 34.4 and 30.7 μm, respectively. Figure 2B and C are the mapping images of Na-MMT and Sr-MMT coatings, respectively, and the distribution of various elements can be clearly seen, which also proves the existence of coating.

**Figure 3.**
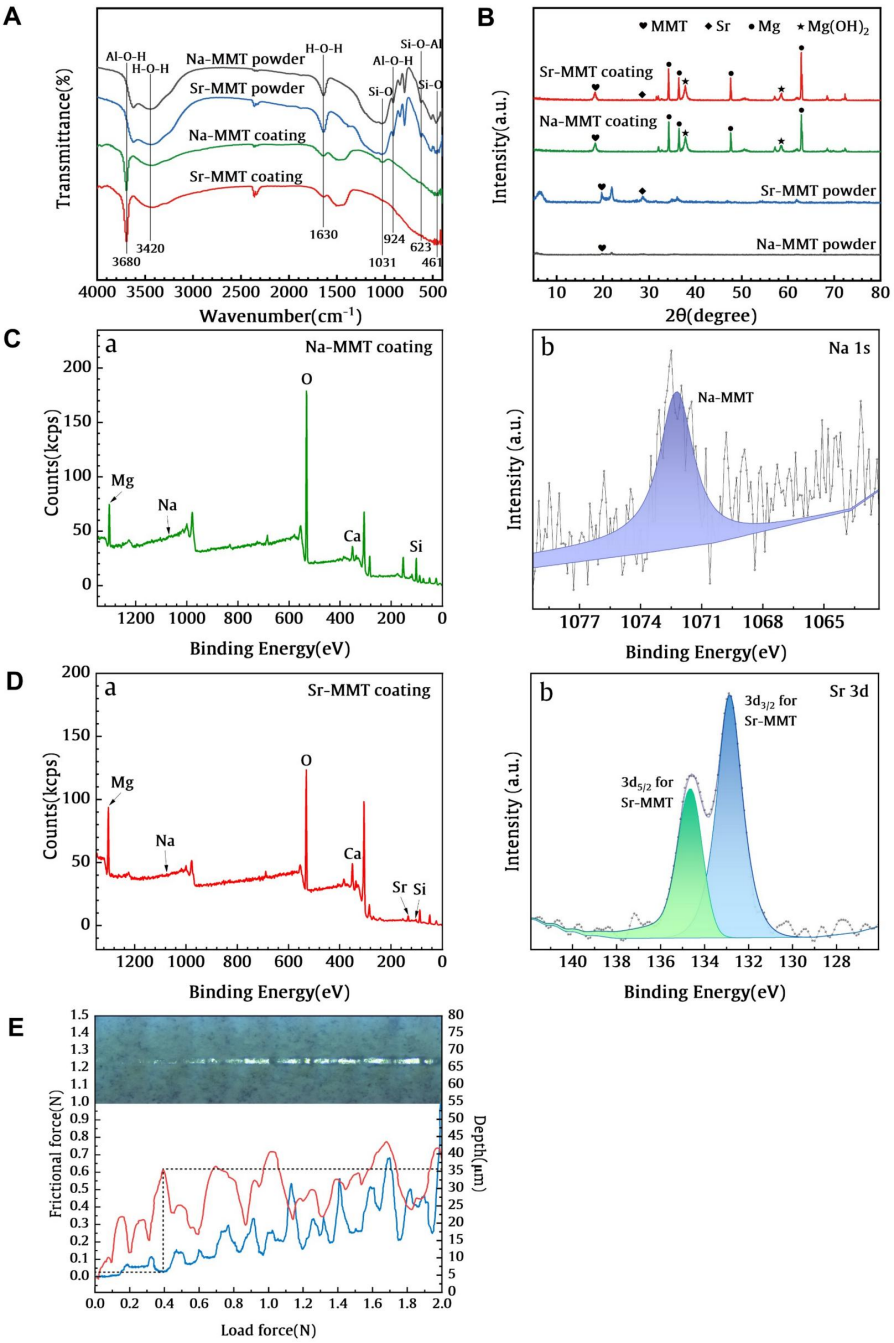
(**A** and **B**) FT-IR Spectra and XRD patterns of the Sr/MMT coating, Sr-MMT powder, MMT coating and MMT powder. (**C**) XPS spectrum of Na-MMT coating (a) and typical peak-fitting results of the Na 1s (b). (**D**) XPS spectrum of Sr-MMT coating (a) and typical peak-fitting results of the Sr 3d (b). (**E**) Frictional force of the Sr-MMT coating.

FT-IR spectroscopy results for Na-MMT and Sr-MMT powder and coating are shown in Figure 3A. It can be seen from the spectrum that there are bending vibration peaks of Si-O at 461 and 1031 cm^−1^, and bending vibration peaks and stretching vibration peaks of Al-OH at 924 and 3680 cm^−1^, which shows that the coating was appropriately produced on the Mg–Ca alloy surface. There is no significant difference in the spectral results of Na-MMT and Sr-MMT coating.

The XRD patterns of the powder and coated samples are shown in Figure 3B. The characteristic peaks of MMT can be seen in all sample spectra at the diffraction angle of 18°; and a tiny Sr characteristic peak of 28° can also be seen on Sr-MMT coating, which shows that the coating is effectively established on the Mg–Ca alloy matrix. Furthermore, Mg peaks are found at 32°, 35°, and 64°, and magnesium hydroxide, Mg (OH)_2_ at 38°and 59°, indicating that the corrosion on Mg–Ca alloy occurred during the reaction.

The peak XPS spectra of Na-MMT and Sr-MMT coating can be seen in Figure 3C and D. It can be seen that both samples have C 1s, O 1s, Ca 2p, Na 1s, Mg 1s, Si 2p. Among them, Si element is the main intensity peak of MMT, and the presence of O element may be Mg (OH)_2_ produced on the surface of Mg–Ca alloy during the hydrothermal reaction, which also corresponds to the XRD result. Moreover, the appearance of Sr element in Figure 3D(b) proves the ion exchange between Sr^2+^ ion and Na^+^ ion, indicating the successful preparation of Sr-MMT coating on Mg–Ca alloy matrix.

The friction force of Sr-MMT coating is shown in Figure 3E. When the load reaches 0.38 N, the scratch depth changes dramatically and penetrates the coating itself with a thickness of 30.7 μm. It means that the coating completely falls off and the binding force of the coating is 0.03 N.

### Corrosion behavior

**Figure 4.**
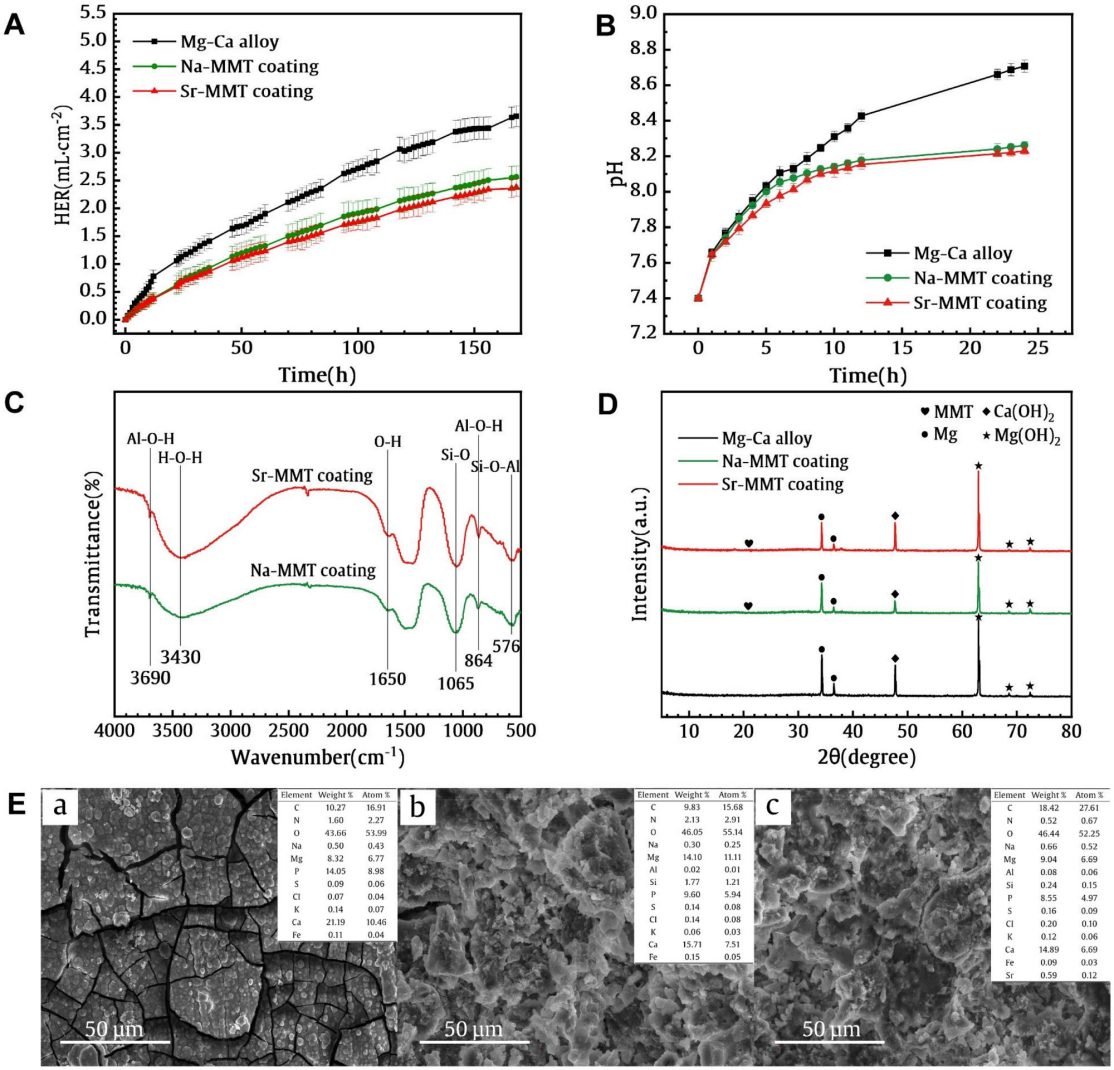
(**A**) HER curves of Mg–Ca alloy, Na-MMT and Sr-MMT coatings immersed for 7 days. (**B**) Change in pH for Mg–Ca alloy, Na-MMT and Sr-MMT coatings in DMEM for 24 h. (**C** and **D**) FT-IR spectra and XRD patterns of the coatings immersed in DMEM for 7 days. (**E**) SEM images and EDS spectra of (a) Mg–Ca alloy, (b) Na-MMT coating and (c) Sr-MMT coating after soaking in DMEM solution for 7 days.

Figure 4A shows the HER of samples immersed in DMEM. The HER of the samples increased sharply at the beginning of the immersion, and then gradually stabilized at 12 h. Moreover, the HER of the Na-MMT- and Sr-MMT-coated alloys were markedly lower than the Mg–Ca substrate. A lower HER indicates a higher corrosion resistance. In other words, the coatings significantly enhanced the corrosion resistance of the Mg–Ca alloy.

The pH changes of the alloy and its coated samples immersed in DMEM are shown in Figure 4B. After 1 day of immersion, the pH of the DMEM solution of the bare alloy was increased up to 8.75, and kept a constant rise. But the pH values for the Na-MMT- and Sr-MMT-coated samples were 8.16 and 8.2, respectively. And then the pH change might remain a steady state.

Figure 4C and D show the FT-IR results and XRD patterns of the samples immersed in DMEM solution for 7 days. The FT-IR results in Figure 4C designate that the characteristic bands of MMT still exist, indicating that the Na-MMT and Sr-MMT coating have a stable performance during the soaking process. In addition, the XRD data shown in Figure 4D shows the same results as Figure 4C. It is noticed that the characteristic peaks of MMT can still be detected on the surface of the immersed coated samples, implying that the coatings are well-bonded with the Mg-Ca substrate.

Figure 4E represents the SEM morphology and EDS spectra of Mg–Ca alloy, Na-MMT coating, and Sr-MMT coating after soaking in DMEM solution for 7 days.

For Mg–Ca alloys, after 7 days of immersion, almost all the corrosion products in the first layer fall off, and there are many matrix cracks. Small cracks appeared on the surface of the Na-MMT coating. After soaking for 7 days, the Sr-MMT coating still exhibits a smooth and dense morphology under SEM. EDS results disclose the presence of Si element on the surface of the two coatings, indicating that the MMT coating still exists on the surface of Mg–Ca alloy. It is worth noting that the EDS results in Figure 4E(a)–(c) also reveal the presence of P element, which may be due to the formation of phosphate and other substances attached to the surface of the material by the phosphate contained in DMEM.

**Figure 5.**
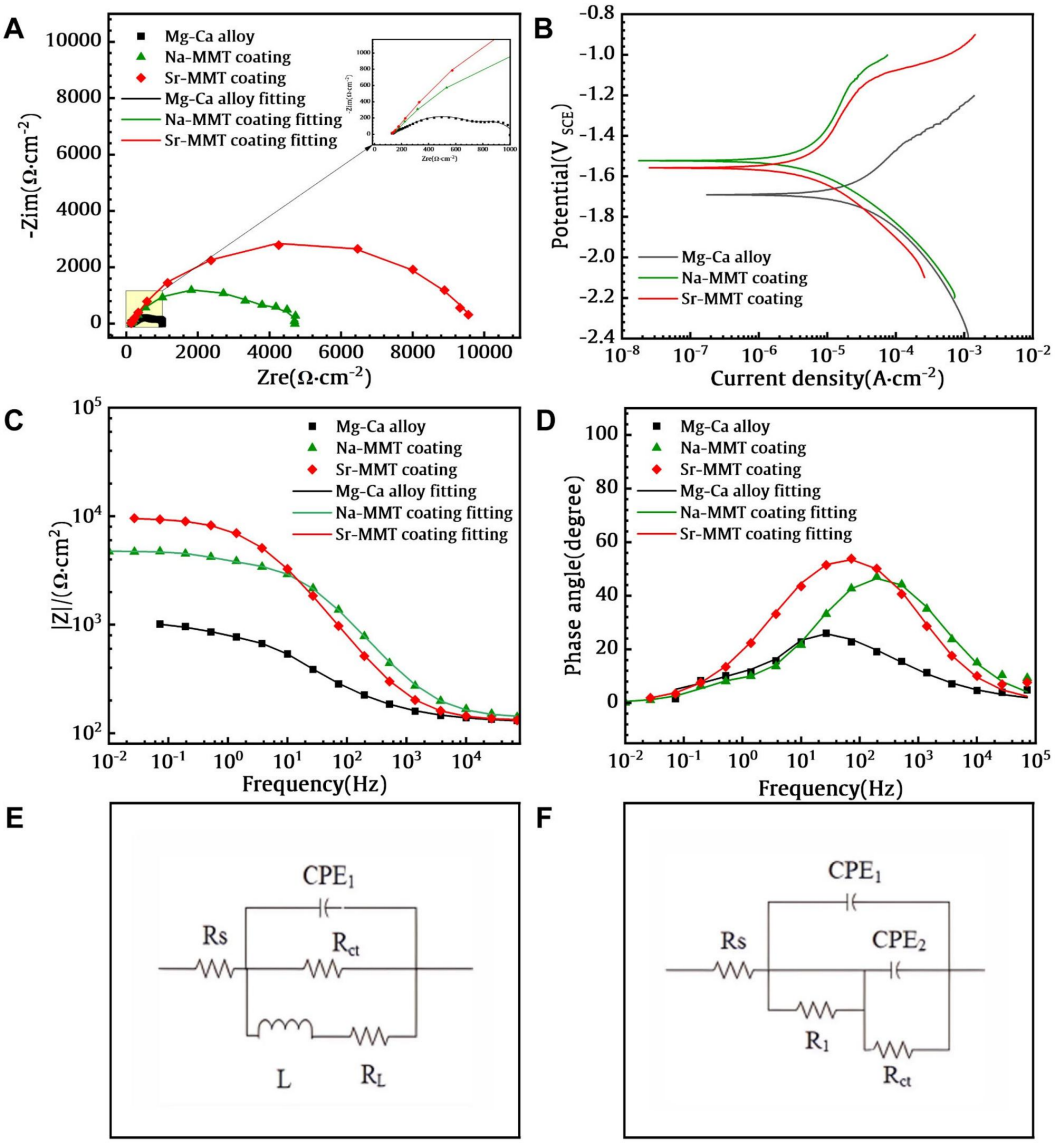
EIS And the fitted results for Mg–Ca alloy, Na-MMT and Sr-MMT coatings: (**A**) Nyquist plots; (**B**) Potentiodynamic polarization (PDP) curves; (**C**) Bode plots of phase angle vs frequency; (**D**) Bode plots of |*Z*| vs frequency in DMEM; (**E**) Equivalent circuits of the Mg–Ca alloy; (**F**) Equivalent circuits of the Na-MMT coating and Sr-MMT coating.

The electrochemical test results are shown in Figure 5. It shows that the Nyquist plots (Figure 5A) of the three samples immersed in DMEM. Mg–Ca alloy exhibits two types of circuits, capacitance circuits in the high-frequency and intermediate frequency ranges, and inductance circuits in the low-frequency range. The formation of capacitive circuits is related to the process of charge transfer, while the formation of inductive circuits is related to the dissolution process and pitting behavior of magnesium alloy matrix in solution. Similar phenomena were observed in the Na-MMT coating samples and Sr-MMT coating samples, with the diameters of the capacitor rings being 4400 and 10 000 Ω·cm^2^, respectively. The two coatings are larger than Mg–Ca alloy (1200 Ω cm^2^), indicating that the preparation of the coating improves corrosion resistance.

Figure 5B demonstrates the potential dynamic polarization curves of three samples in DMEM solution, and the curves are fitted to calculate the parameters. The corrosion potential (*E*_corr_) and corrosion current density (*I*_corr_) are derived from the polarization curves and recorded in Table 1. In theory, the lower the corrosion current density of a material, the better its corrosion resistance. The *I*_corr_ of Na-MMT and Sr-MMT coatings are 3.70 × 10^−6^ A·cm^−2^ and 3.97 × 10^−6^ A·cm^−2^, much lower than bare Mg–Ca alloy (1.76 × 10^−5^ A·cm^−2^), indicating that both coatings have better corrosion resistance than Mg–Ca alloy substrate. In comparison to Mg–Ca alloy, the Sr-MMT coating exhibits a positive potential change of 140 mV, indicating that Sr-MMT coating has better corrosion resistance.

**Table 1.** Electrochemical parameters of polarization curve

Samples	*E*_corr_ (*V*_SCE_)	*I*_corr_ (A cm^−2^)
Mg–Ca alloy	−1.69	1.76 × 10^−5^
Na-MMT coating	−1.52	3.70 × 10^−6^
Sr-MMT coating	−1.55	3.97 × 10^−6^

In general, at lower frequencies, a higher |*Z*| modulus means higher corrosion resistance. As shown in Figure 5C and D, the |*Z*| modulus of Sr-MMT coating alloy is 7.2 × 10^4^ cm^2^ at low frequencies, which is significantly higher than Mg–Ca alloy. Moreover, at lower frequencies, the Sr-MMT coating has a high and wide phase angle, it reflects the uniform density of the coating, and has a valid protective effect on the Mg–Ca alloy matrix.

Due to the presence of coatings, there are differences in the circuit conditions between the two coating samples and Mg–Ca alloy under applied current. Figure 5E and F show the fitting circuit diagrams of the three samples. *R*_s_ and *R*_ct_ are solution resistance and charge transfer resistance, and inductance is represented by *L*. CPE_1_ and CPE_2_ are used to represent capacitors. The fitting circuit of the Mg–Ca alloy substrate is shown in Figure 5E. CPE_1_ and R_1_ represent high-frequency capacitor circuits, representing the corrosion product layer formed on the surface of the Mg–Ca alloy. In the inductance circuit, the appearance of *R*_L_ and *L* indicates the pitting corrosion process of Mg–Ca alloy. The fitting circuit of Na-MMT coating and Sr-MMT coating is shown in Figure 5F. The presence of two capacitors in the circuit indicates that the corrosion behavior of the two coating samples is composed of double-layer resistors. They are the film layer between the coating and the solution, and the product film layer formed by the reaction between the coating and the surface of the Mg–Ca alloy. Although Na-MMT coating and Sr-MMT coating can be fitted using the same circuit, there are differences in *R*_1_ and *R*_ct_ between the two samples. From the fitting data results in Table 2, it can be seen that the *R*_1_ and *R*_ct_ of Sr-MMT coating are higher than Na-MMT coating, and the *R*_1_ of both coatings are higher than that of Mg–Ca alloy. The results indicate that the preparation of the coating can provide protection for the substrate, and the Sr-MMT coating has better corrosion resistance.

**Table 2.** Equivalent circuit fitting data of EIS curves

Samples	*R*_s_, Ω·cm^2^	CPE_1_, Ω^−1^ cm^−2^ s^n^	*n*_1_	*R*_1_, Ω·cm^2^	CPE_2_, Ω^−1^ cm^−2^ s^n^	*n*_2_	*R*_ct_, Ω·cm^2^	*R*_L_, Ω·cm^2^	*L*, H·cm^2^	Χ^2^
Mg–Ca alloy	125.7	2.55 × 10^−4^	0.5	–	1.69 × 10^−3^	0.94	1827	2193	69.57	0.0008
Na-MMT coating	137.5	8.27 × 10^−6^	0.71	3849	4.85 × 10^−4^	0.93	778.1	–	–	0.0004
Sr-MMT coating	130.3	1.22 × 10^−5^	0.73	8651	2.70 × 10^−4^	0.82	863.6	–	–	0.0006

### *In vitro* study

#### Cytotoxicity assay

**Figure 6.**
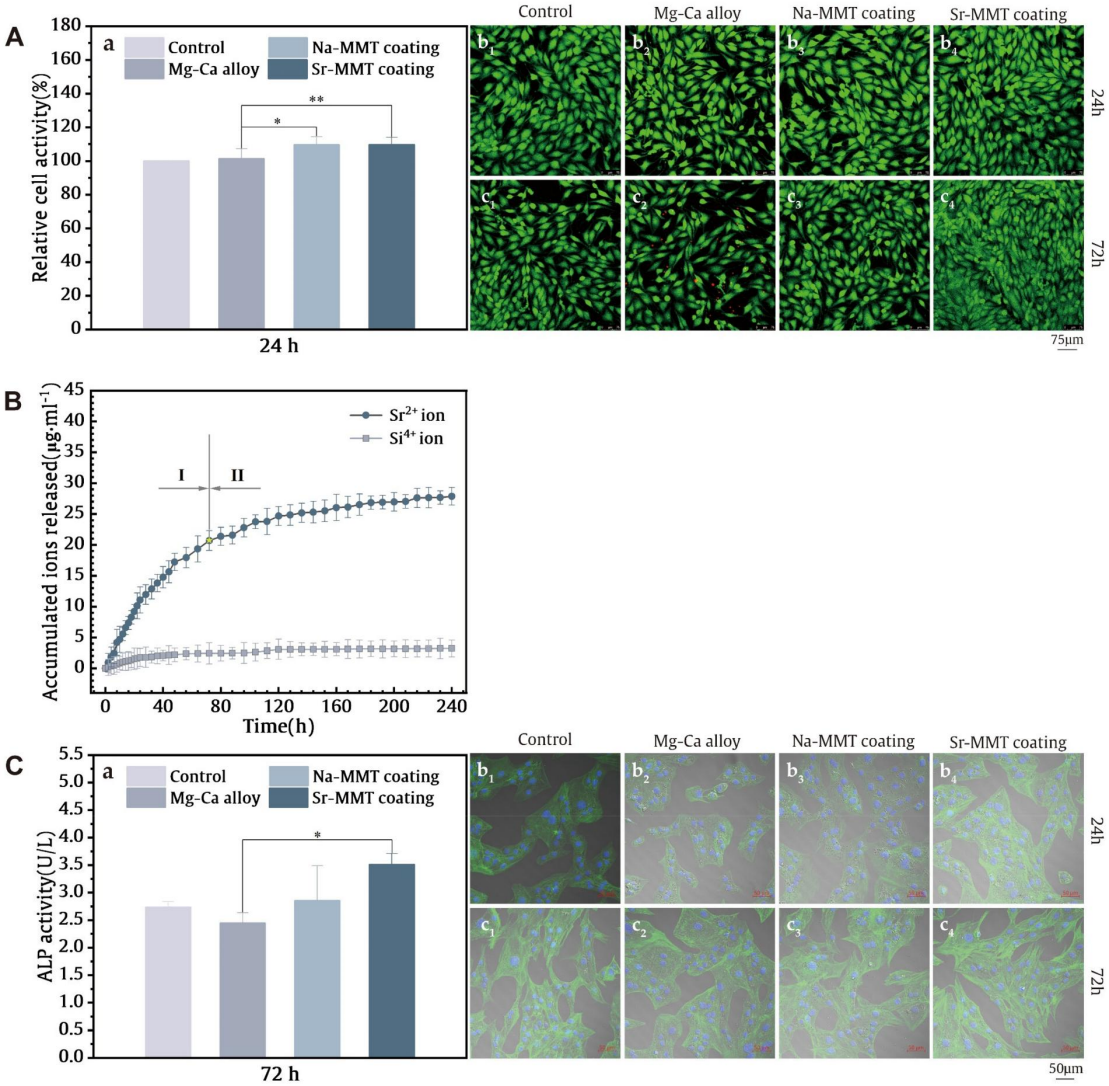
(**A**) Relative activity of MC3T3-E1 cells incubated for 24 h (a); images of live/dead staining incubated for 24 h (b_1_–b_4_) and 72 h (c_1_–c_4_) (*P ＜ 0.05). (**B**) Sr^2+^ and Si^4+^ release curve of Sr-MMT immersed in DMEM solution for 10 days. (**C**) Viability of ALP when Mg–Ca alloy, Na-MMT and Sr-MMT coatings were co-cultured with MC3T3-E1 cells for 72 h (a), CLSM shooting results (200×) after the sample was co-cultured with MC3T3-E1 cells for 24 h(b_1_–b_4_) and 72 h(c_1_–c_4_) (*P ＜ 0.05).

Similar to the MMT method, CCK-8 can be used for the analysis of cell proliferation and toxicity, as shown in Figure 6A(a). The detection principle is that under the action of an electronic carrier, intracellular dehydrogenases can reduce WST-8(2-(2-methoxy-4-nitrophenyl)-3-(4-nitrophenyl)-5-(2,4-disulfobenzene)-2H-tetrazole monosodium salt) contained in CCK-8 reagent, generating highly water-soluble yellow formazan product. The amount of formaldehyde produced is positively correlated with the number of living cells. After 24 h of culture, the relative cell viability of the Mg–Ca alloy, Na-MMT and Sr-MMT coatings were 101.4%, 109.6% and 109.7%, respectively. The Na-MMT and Sr-MMT coatings showed higher cell viability (*P* < 0.05). It is believed that the corrosion behavior of the sample in the leaching solution may change the pH of the leaching solution, thereby affecting the cell viability. The coating slows down the corrosion rate of magnesium alloy matrix, resulting in a slowing down of the alkalization rate around the matrix, which weakens the damage to cells to a certain extent. In general, bare Mg–Ca alloy itself has good biocompatibility, and the alloy material covered by coating also has preferable biocompatibility (ISO-10,993:5).

#### Live/dead cell staining

Figure 6A(b) and (c) exhibits the live and dead cell staining results. Live cells are shown in green and dead cells red in the images. Compared with the control group, there was no obvious abnormality in the three groups of samples after culturing for 24 h. While, after 72 h of culture, red dots could be clearly observed in the pictures of the Mg–Ca alloy group, which indicated the existence of dead cells. It may be that the pH changed around the alloy after corrosion greatly, making it difficult for some cells to survive. It can be seen that the results observed under the laser confocal microscope are basically consistent with the relative cell activity. It proves that the Na-MMT and Sr-MMT coating have good biocompatibility.

#### Osteogenic performance assay

The cumulative release curve of Sr^2+^ and Si^4+^ in the Sr-MMT coating in DMEM solution is shown in Figure 6B. The release process of Sr^2+^ shows different regularities in two different stages, during the first 80 h of immersion, the release rate of Sr^2+^ is faster, and the content of Sr^2+^ in the solution accumulates rapidly (the first stage). Then, the release rate begins to decrease, gradually stabilizes, and finally, the hourly release amount significantly decreases (the second stage). The change in Sr^2+^ content is not significant after soaking for 200–240 h, and the Sr^2+^ in the coating may have been completely released or the content is relatively low. The concentration of Sr^2+^ in DMEM solution after 10 days of final soaking is 29 μg/ml. Si^4+^ was released slowly and steadily, and the final concentration reached 3.22 μg/ml.

Alkaline phosphatase (ALP) is mainly synthesized and secreted by the liver and osteoblasts, and is the most important indicator of bone metabolism. The ALP results are shown in Figure 6C(a). The ALP activities of the Na-MMT group (2.8 U/l) and the Sr-MMT group (3.5 U/l) are higher than the bare Mg–Ca alloy group (2.4 U/l), and the Sr-MMT group is significantly higher than the bare alloy group(*P* < 0.05). The results show that the Sr-MMT coating can promote the formation of ALP.

The cytoskeleton protein changes of MC3T3-E1 after 24 and 72 h of culture under the CLSM are shown in Figure 6C(b) and (c). The cells were looser and had no obvious pseudopodia after 24 h of culture. And after 72 h of incubation, the spreading area of the cells increased, the number of nuclei in the area increased, the pseudopodia were elongated, and the connection between cells was tighter. It means that the changes of Sr-MMT group are more obvious than other groups. The incorporation of strontium element promotes the growth of bone cells, so the Sr-MMT coating shows more obvious cell extension.

### *In vivo* osteogenesis performance

#### X-ray image analysis

**Figure 7.**
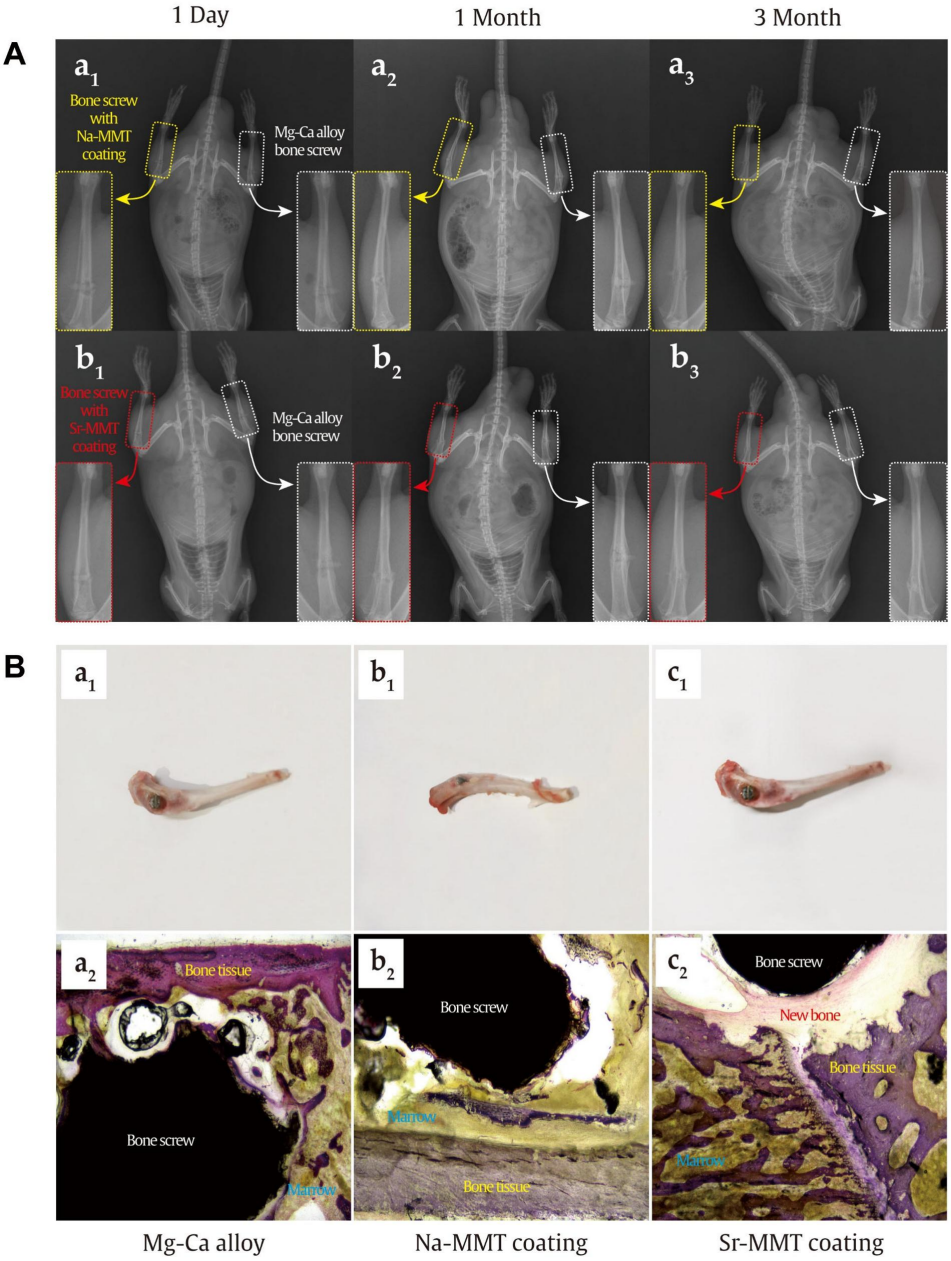
(**A**) X-ray films of rats implanted with bone screw for 1 day, 1 month and 3 months: Na-MMT group (a_1_–a_3_) and Sr-MMT group (b_1_–b_3_). (**B**) HE staining images after hard tissue section: Mg–Ca alloy (a_1_ and a_2_), Na-MMT coating (b_1_ and b_2_) and Sr-MMT coating (c_1_ and c_2_). black areas: implanted bone screw; purple areas: bone tissue; dark areas: bone tissue; yellow: marrow; red areas: new bone.

Figure 7A presents the X-ray films of rats taken on the day of bone screw implantation and 1 month later. In pictures, the left tibia of rats is implanted with Mg–Ca alloy bone screws, and the right tibia is implanted with Na-MMT-coated bone screws (a) and Sr-MMT-coated bone screws (b), respectively. We can see from Figure 7A(a1) and (b1) that on the first day of implantation, the original bone tissue of the proximal knee joint of the rat tibia was damaged, the defect site was broken around and there was a gap between the implant and the defect site. One month after the bone screw was implanted, the periosteal effect occurred in the left leg tibia of rats, and the bone diameter increased significantly. This condition did not occur in the right leg of rats, and bone tissue began to grow at the implant site to a certain extent under the effect of the two coatings. As can be seen in Figure 7A(a3) and (b3), after 3 months of culture of bone defect model rats, the periosteal reaction of the original left leg tibia has disappeared. In Figure 7A(b3), the regenerated bone tissue at the defect site of the right leg of the rat has been completely coated with Sr-MMT-coated bone screws. Because of the biodegradability of magnesium alloy, the volume of bone screws in the two groups of experiments has changed to a certain extent.

**Figure 8.**
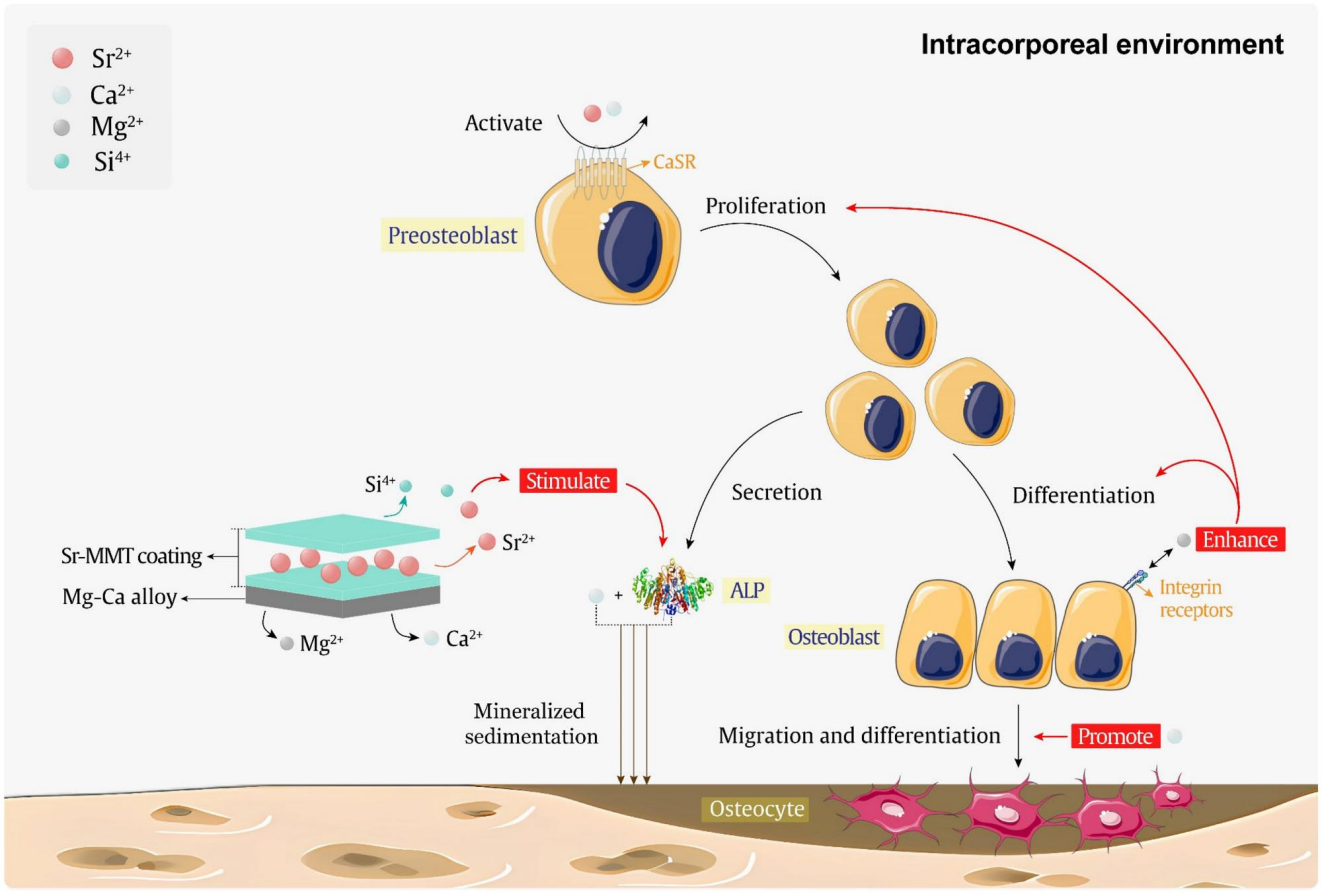
Mechanism of promoting bone formation.

#### Hard tissue section and staining

Figure 7B shows HE staining images after hard tissue section. Figure 7B(a) and (b) show the bare alloy sample and the sample covered with Na-MMT coating, respectively, it can be seen that the gap between the broken bone tissue around the implant and the bone tissue is large, and the combination with the bone tissue is weak. Vacuoles can be seen in the pictures of the bare alloy sample. The shape of the surrounding bone tissue is basically normal, and inflammatory cells infiltrate locally. Around the implant, new bone trabeculae can be seen stretching from the implant to the matrix bone. Figure 7B(c) shows the Sr-MMT coating sample picture, it can be seen that the implant is closely combined with bone tissue, the surrounding bone tissue morphology is normal, the bone trabecula in the matrix bone grows well, the osteoblasts are linear around the bone trabecula, and there is a clear transition between the growth of collagen fiber cells around the implant and the bone trabecula in the matrix bone.

## Discussion

In the design process of bone screws, we did not use different countermeasures to solve different problems one by one, but chose a more flexible design idea, cleverly combining Mg–Ca alloy, MMT and Sr^2+^, following the principle of promoting strengths and avoiding weaknesses, striving to maximize the performance of materials. Mg–Ca alloy has excellent biosafety and mechanical properties, it degrades without releasing metal ion and acidic substances that are harmful to the growth of new bone. MMT is a body-friendly carrier and is often used as a drug carrier [53–55] and cell tissue scaffold [56–58]. MMT can not only act as a strong physical barrier to delay the corrosion of the alloy matrix, but also because of its unique lamellar structure, so that the stent has a slow continuous Sr^2+^ release ability, which solves the problem of drug burst release in the drug-loaded coating. Therefore, after making full use of the characteristics of various substances, an efficient and comprehensive ability to promote bone were achieved.

### Influence of simulated body fluid

The selection of simulated body fluid also has certain influence on the results of corrosion resistance test. For implant materials, to better simulate the human body environment, such as SBF, PBS, Hank’s are often used as the simulated body fluid for corrosion resistance test, in which SBF is the most widely used.

However, studies have shown that simple salt solution is not suitable for the determination of corrosion resistance of implant materials. Moreover, Mg–Ca alloy samples may form calcium carbonate clusters in SBF, which may affect the test results. In this case, DMEM solution used as cell culture medium is a good choice for corrosion solution because it has similar ion concentration with body fluid and has amino acids, glucose and other nutrients.

Table 3 lists and compares the corrosion resistance test data of several samples coated with inorganic coating on the surface of Mg–Ca alloy under different corrosion solutions. Dilara *et al*. [59] prepared gallium-doped Ca-P coating on the surface of Mg–Ca alloy, and selected the same corrosion solution as this study in the corrosion resistance test, greatly improving the corrosion potential and corrosion current density of the original Mg–Ca alloy. The coatings prepared in this study showed similar corrosion resistance results. Zhu *et al*. [60] have shown that the presence of glucose and amino acids has a good effect of slow corrosion, and can promote the formation of metal complexes on the surface of the sample, thus slowing down the metal corrosion. Compared with that using SBF as corrosion solution [61–63], the *E*_corr_ of Sr-MMT coating is higher, and the *I*_corr_ is lower. It is indicated that the preparation of Sr-MMT coating improves the corrosion resistance of Mg–Ca alloy. And because the composition of DMEM is closer to the human body, our results can better reflect the corrosion of metal materials in the human body. In addition, our previous work also studied the corrosion resistance of the MMT coating on the AZ31 surface. Both MMT-BSA coating and GS-MMT coating show lower corrosion current density than bare AZ31 [38, 45]. It shows that the selection of MMT coating materials has a certain effect on the corrosion resistance of magnesium alloys.

**Table 3.** Corrosion behavior of bio-coatings in different corrosion solutions

Material	Coating	Solution	Bare alloy		Coating		References
			*E*_corr_ (*V*_SCE_)	*I*_corr_ (A/cm^2^)	*E*_corr_ (*V*_SCE_)	*I*_corr_ (A/cm^2^)	
Mg–Ca	Sr-MMT	DMEM	−1.69	1.76 × 10^−5^	−1.55	3.97 × 10^−6^	–
Mg–Ca	CaP	DMEM	−1.8	2.18 × 10^−5^	−0.28	6.5 × 10^−8^	[59]
Mg–Ca	CaP	SBF	−1.63	9.00 × 10^−5^	−1.46	4.10 × 10^−6^	[61]
Mg–Ca	RKKP	SBF	−1.86	5.75 × 10^−4^	−1.68	6.13 × 10^−5^	[62]
Mg–Ca	HA-PCL	Kokubo	−1.68	2.68 × 10^−4^	−1.27	5.85 × 10^−8^	[64]
AZ31	GS-MMT	DMEM	−1.44	1.98 × 10^−5^	−1.35	1.67 × 10^−6^	[38]
AZ31	MMT-BSA	Hank’s	−1.41	6.27 × 10^−6^	−1.4	7.65 × 10^−7^	[45]

### Mechanism of strontium MMT coating promoting bone growth

The ability of Sr-MMT coating to promote bone formation was demonstrated through *in vitro* ALP activity measurement and the establishment of bone screw animal models. This may be the result of the combined action of a small amount of Mg^2+^ and Ca^2+^ dissolved during the degradation process of Mg–Ca alloy with Si^4+^ and Sr^2+^ released from the Sr-MMT coating, as shown in Figure 8.

First, it is well known that metal implants dissolve metal ions when they enter the body, and Mg–Ca alloys with Sr-MMT coatings are no exception. They can dissolve small amounts of Mg^2+^ and Ca^2+^ both *in vivo* and *in vitro*. Mg^2+^ can interact with integrin receptors on the membrane surface of osteoblasts or osteoclast to enhance the proliferation, differentiation and adhesion of preosteoblasts. Extracellular Ca^2+^ can indirectly affect bone remodeling by altering levels of parathyroid hormone and vitamin D3 [65]. However, recent studies have shown that bone cells can also directly perceive and respond to Ca^2+^ levels. Calcium sensing receptor (CaSR) is a protein-coupled receptor (GPCR) located in bone cells and has been found to be associated with bone remodeling. Moreover, low concentration of Ca^2+^ is conducive to osteoblast migration, and high concentration of Ca^2+^ is conducive to osteoblast differentiation [66].

Second, the immersion test results of Sr-MMT coating in DMEM showed that Sr^2+^ was slowly released and the cumulative release of strontium ions after 10 days of immersion was 29 μg/ml. In addition, MMT in the coating is a drug carrier containing Si^4+^, and we also demonstrated the micro-release of Si^4+^ ions in DMEM solution. ALP is an early marker of osteogenic differentiation, and its expression and activity levels decrease as osteoblasts mature. Relevant studies have proved that Sr and Si have synergistic effects in promoting bone mineral formation. And ALP can hydrolyze phosphate esters. It can increase the reaction between phosphate and Ca^2+^ and promote mineralization of collagen matrix in bone tissue. Mao *et al*. [67] investigated the expression of ALP in cells treated with Si and Sr ions alone and in combination with the same concentration of Si and Sr ions. They found that although both ions had a stimulating effect on ALP expression when used alone, the combination of the two ions produced the greatest effect. Similarly, the combination of the two ions has the greatest inhibitory effect on the production of osteoclast. At the same time, CaSR can also respond to the binding of other cations such as Sr^2+^, stimulating osteoblasts to promote the expression of bone-related genes [68]. Li *et al*. [69] also confirmed the promotion of Sr^2+^ on ALP expression when strontium at different concentrations induced the activity of extracellular Viral matrix protein ALP. This also corresponds to the experimental results of this study. The ALP activity of Sr-MMT coating group was 3.5 U/l. The synergistic effect of Si^4+^ and Sr^2+^ in strontium-doped Na-MMT coating significantly promoted the expression of ALP. Therefore, the preparation of strontium-doped Na-MMT coating can improve the bone repair performance of Mg–Ca alloy.

Third, in the bone cell proliferation experiment, both the Mg–Ca alloy group and the coating group showed a decrease in cell viability. The reason may be that during *in vitro* cell culture in cell culture medium, Mg–Ca alloy undergoes degradation, leading to an increase in the pH value of the culture medium, which is not conducive to cell growth. However, after the Mg–Ca alloy bone screw is implanted into the body, the surrounding tissue of the implant is bone tissue rather than solution, and the subsequent surrounding connective tissue wrapping can only cause local inflammation, which has been confirmed in tissue slices and X-ray films.

Despite careful consideration of many key factors, there are limitations to this study. First, the corrosion resistance of the material is not the higher the better, and its degradation rate should match the fracture healing time, so the release of strontium and the degradation of the alloy need to be further accurately regulated. In addition, in order to achieve future clinical application, it is also necessary to further extend the observation period after implantation in rats until the alloy is completely degraded, and explore the long-term impact of the material on the body.

## Conclusions

In the study, we demonstrated the Sr-MMT coating with slow-release and bone promoting properties to address the issues of Mg-based implant corrosion and service life. The Sr-MMT coating was prepared via hydrothermal method. The main conclusions are as follows:

1. The results of immersion and electrochemical corrosion experiments indicate that Sr-MMT coating can improves the corrosion resistance of Mg–Ca alloy.
2. After cocultivation for 72 h, the relative cell activity of MC3T3-E1 was 104.5%. In the images of live/dead cell staining experiments, the Mg–Ca alloy group showed the highest number of dead cells. The experimental results indicate that Sr-MMT coating can enhance the biocompatibility of Mg–Ca alloy.
3. The ALP activity results showed that the activity of the Sr-MMT coating group (3.5 U/l) was significantly higher than that of the Mg–Ca alloy group (2.4 U/l). After 90 days of *in vivo* implantation experiments, it can be seen that the Sr-MMT coating has a better effect on promoting bone tissue generation and promoting the integration of the implant with surrounding bone tissue. The results indicate that the Sr-MMT coating has good bone promoting properties.

## Supplementary Material

rbae027_Supplementary_Data
