# Supplementary material for: In vitro and in vivo degradation, biocompatibility and bone repair performance of strontium-doped montmorillonite coating on Mg–Ca alloy
Source: Regen Biomater. 2024 Mar 22;11:rbae027. doi: 10.1093/rb/rbae027 (PMC11007119; doi:10.1093/rb/rbae027)
Supplement: rbae027_Supplementary_Data [file rbae027_supplementary_data.zip › The ethical statement.pdf]

青岛大学附属医院医学伦理委员会审批件

伦审批件号：QYFY WZLL 32751

项目名称：镁钙合金表面掺锶蒙脱石涂层体内外降解、生物相容性及骨活性研究

承担单位：青岛大学附属医院

项目负责人：任延德

项目简介：本课题在镁钙(Mg-Ca)合金表面制备掺锶蒙脱石(Sr-MMT)涂层，首先对其进行表征及耐蚀性分析，选用 MC3T3-E1 细胞作为研究对象，通过细胞毒性实验和活死细胞染色研究涂层的体外生物相容性，最后建立大鼠胫骨植入模型探讨对其体内生物相容性及成骨性能。实验结果显示涂层能够显著提高镁钙合金的耐蚀性能、生物相容性及成骨性能，为生物医用镁合金的临床应用提供了理论基础。

医学伦理委员会意见：

该计划项目中，受试者权力和利益得到充分保护，符合医学伦理委员会要求。同意研究方案。

医学伦理委员会(盖章)：

2023 年 10 月 24 日
