# Supplementary material for: In vitro and in vivo degradation, biocompatibility and bone repair performance of strontium-doped montmorillonite coating on Mg–Ca alloy
Source: Regen Biomater. 2024 Mar 22;11:rbae027. doi: 10.1093/rb/rbae027 (PMC11007119; doi:10.1093/rb/rbae027)
Supplement: rbae027_Supplementary_Data [file rbae027_supplementary_data.zip › 2022.6.10-The ethical statement.pdf]

青岛大学附属医院医学伦理委员会审批件

伦审批件号：QYFY WZLL 32751

项目名称： 可降解镁合金表面蒙脱石锶涂层的制备及骨活性研究

承担单位： 青岛大学附属医院

项目负责人： 任延德

项目简介： 本课题在可降解镁钙(Mg-Ca)合金表面制备蒙脱石锶(Sr-MMT)涂层，研究内容：(1)以硝酸锶 ($Sr(NO_3)_2$)、Na-MMT 为原料，以 Sr^{2+} 含量为检测指标，采用离子交换法制备 Sr-MMT，首先进行单因素实验，研究硝酸锶的量、温度、时间对 Sr-MMT 中锶含量的影响；然后，在单因素基础上，利用正交法优化工艺条件。分析锶插层蒙脱石的机理。(2)利用水热法，在 Mg-Ca 合金表面制备 Sr-MMT 涂层；利用电镜、EDS、XRD 及 FTIR 等观察涂层表面及纵切面形貌其结构，分析组成；以 DMEM 为模拟体液，利用浸泡后电镜观察、析氢实验等探讨 Sr-MMT 涂层降解机理，涂层与金属结合力的强弱。 (3)通过溶血率、动态凝血时间等实验研究涂层的体外血液相容性；通过细胞毒性试验，碱性磷酸酶测定等探讨 Sr-MMT 涂层中锶诱导骨活性的机理。 (4)建立动物植入模型，研究 Sr-MMT 涂层的 Mg-Ca 合金体内降解情况及对机体的影响。该项目实验动物模型的建立部分以 Wistar 大鼠为实验动物，将 Sr-MMT 涂层骨钉植入胫骨处，定期观察骨钉降解及骨生长情况，3 月后局部取出做硬组织切片，观察骨钉与骨及周围组织状况。

医学伦理委员会意见：

该计划项目中，受试者权力和利益得到充分保护，符合医学伦理委员会要求。同意研究方案。

医学伦理委员会(盖章)：

2022年 6月 10日
